# Understanding help-seeking amongst university students: the role of group identity, stigma, and exposure to suicide and help-seeking

**DOI:** 10.3389/fpsyg.2015.01462

**Published:** 2015-09-29

**Authors:** Michelle Kearns, Orla T. Muldoon, Rachel M. Msetfi, Paul W. G. Surgenor

**Affiliations:** ^1^Department of Psychology, University of LimerickLimerick, Ireland; ^2^Pieta House, Centre for the Prevention of Suicide and Self-HarmLucan, Ireland

**Keywords:** help-seeking, mental health, suicide, group identification, stigma, university students

## Abstract

**Background:** Despite a high prevalence of suicide ideation and mental health issues amongst university students, the stigma of help-seeking remains a barrier to those who are in real need of professional support. Social identity theory states that help received from an ingroup source is more welcome and less threatening to one's identity than that from a source perceived as outgroup. Therefore, we hypothesized that students' stigma toward seeking help from their university mental health service would differ based on the strength of their identification with the university.

**Method:** An online survey including measures of stigma of suicide, group identification, experience with help-seeking and exposure to suicide was administered to Irish university students (*N* = 493).

**Results:** Group identification was a significant predictor of help-seeking attitudes after controlling for already known predictors. Contrary to our expectations, those who identified more strongly with their university demonstrated a higher stigma of seeking help from their university mental health service.

**Conclusions:** Results are discussed in relation to self-categorization theory and the concept of normative fit. Practical implications for mental health service provision in universities are also addressed, specifically the need for a range of different mental health services both on and off-campus.

## Introduction

Seeking help is essential if people are to access appropriate mental health services. While there are different sources of help, both formal and informal (Rickwood et al., 2005), there is acknowledged value in seeking formal help, in particular talking therapies and psychological services (NICE, 2004). It is therefore essential that people feel they can access them. In universities, amongst a constituency that is often perceived as having a high risk of suicide (Borges et al., 2010), these services are seen as an important arm of general pastoral care and student support (Bishop, 1990; Kitzrow, 2003). Indeed provision of services that are close, on campus are viewed as a key spend in these difficult financial times. For this reason the current study looks at help-seeking in students and how it is affected, negatively by social forces such as stigma as well as positively by a sense of belonging at university.

Students are an important group for the study of help-seeking. They have high prevalence rates of mental health problems and suicidal ideation. For example, one web-based study of 763 students showed that around one-third were experiencing a mental health problem at the time of test, of which 60% were present 2-years later (Zivin et al., 2009). In addition, a large survey of over 26,000 students in the US, showed that 18% of undergraduates had seriously considered taking their own lives (Drum et al., 2009). Commentators suggest numerous reasons for this trend including the stressors and pressures of student life, and the adjustment to life away from home and family (Furr et al., 2001), with risk factors including financial difficulty, poverty, substance abuse, sexual victimization, and issues related to sexual identity (Eisenberg et al., 2007). However, many students with mental health problems, particularly suicidal ideation, do not seek help from formal sources of support such as university counseling services or mental health services (Drum et al., 2009; Hunt and Eisenberg, 2010). This is despite the fact that these are often the most accessible forms of support for students, particularly for those living away from home.

The single most commonly cited barrier to professional help-seeking is stigma (Corrigan, 2004; Vogel et al., 2009). Mental health stigma can be conceptualized as a set of negative attitudes that represent prejudice or negative stereotypes about people with mental ill health, and in some cases can lead to significant discrimination (Corrigan and Penn, 1999; Corrigan, 2004; Masuda and Latzman, 2011). Stigma toward mental health is generally associated with negative help-seeking attitudes (Leong and Zachar, 1999; Vogel et al., 2005), and this has been shown to be particularly true for people who are experiencing suicidal thoughts (Rickwood et al., 2005; Batterham et al., 2013). Research demonstrates that this may potentially be due to less openness to and a lower perceived value of professional treatment amongst those with a higher stigma of mental health (Coppens et al., 2013). Previous research has also highlighted a desire for social distance from people with mental health issues or seeing mental ill health as a personal weakness as being related to a reluctance to seek professional help (Schomerus et al., 2009; Mojtabai, 2010; Griffiths et al., 2011).

There has been increasing recognition in recent years for the need to focus on reducing the stigma surrounding suicide in order to positively influence help-seeking attitudes amongst at-risk individuals, something that has been highlighted by the National Action Alliance for Suicide Prevention's Research Prioritization Task Force in their 2014 prioritized research agenda (Pearson et al., 2014). Evidence to date has shown that such strategies may be successful; a suicide prevention initiative implemented within a university setting successfully reduced the stigma of mental health problems and improved students' attitudes toward talking about mental health problems and suicide (Pearce et al., 2003). Stigma is far from the only factor influencing help-seeking behaviors however with a growing body of evidence detailing how it is not just attitudinal but also experiential variables that may impact upon how professional help is viewed.

Past experience with help-seeking has consistently been shown to influence attitudes toward help-seeking for mental health and suicide. These may be either personal experiences (e.g., Rickwood et al., 2005), or knowing someone else that has previously engaged with mental health professionals. Research suggests that attitudes toward formal support services for mental health and suicide are at least partially transmitted through an individual's social network, which also plays a vital role in determining whether a person makes the choice to seek professional help (Rickwood and Braithwaite, 1994; Angermeyer et al., 1999; Vogel et al., 2007). For example, university students who actually sought mental health services knew someone else who had sought help 92–95% of the time, and those who knew someone who had previously sought help had more positive attitudes toward mental health services (Vogel et al., 2007). Of course where these experiences have been negative they can have the opposite effect. A review focusing on determinants of help-seeking amongst young people experiencing issues with suicide found that when past experiences of seeking help were negative, particularly when the young person felt they were not helped or that their problems weren't taken seriously, they acted as substantial barriers to future help-seeking intentions and impacted heavily upon attitudes toward professional help (Rickwood et al., 2005).

Help-seeking attitudes may also be influenced by past exposure to suicide or experience with suicidal behaviors. Whilst some studies found no significant relationship between exposure to suicide and help-seeking attitudes or intentions (Calear et al., 2014), others found that exposure to suicide led to more negative attitudes toward help-seeking (Chan et al., 2014). Moreover, research also suggests that attitudes may differ dependent on the amount of exposure. Just 5% of Irish men would turn to a mental health professional as a source of support for suicide if they had no previous exposure to suicide. This increased to 19% when they knew one person who died by suicide but dropped to 8% again when they knew of more than one suicide (Begley et al., 2004).

Therefore, whilst it seems that this factor may influence attitudes toward help-seeking, the way in which this relationship works has not yet been fully established. On the one hand it is thought that past experience with help-seeking may act as a form of knowledge or mental health literacy which is deemed important in the help-seeking process (Gulliver et al., 2010; Coppens et al., 2013). Students who know someone that have accessed mental health services and that have positive expectations about how friends and family would think of them if they sought this type of service professional help are more likely to have positive attitudes to help-seeking (Gulliver et al., 2010; Coppens et al., 2013). These findings implicate shared attitudes and feelings of belonging, two important components of social identity, in determining help-seeking behavior which is entirely consistent with recent research which has pointed to the importance of shared social identities in determining access and availability to social support (Haslam et al., 2005).

Recent empirical evidence demonstrates that where individuals share group membership, they are more likely to provide each other with support, receive support, and interpret support offered in the manner in which it is intended, in comparison to those where shared membership is absent (Reicher et al., 2006). However, the capacity for this social support to affect appraisal depends on the match between group membership of the support provider and recipient. So support does not always have the same or equivalent impact (Haslam et al., 2005). It seems to vary systematically as a function of the group membership of the support provider. For example, university students who were informed that a task was challenging rather than stressful appraised the task more positively and demonstrated less cardiac reactivity when the information provided came from an ingroup member rather than an outgroup member (Haslam et al., 2004; Gallagher et al., 2014; For a review of the potentially harmful effects that intense emotions, particularly stress, can have on cardiovascular health, see Steptoe and Kivimäki, 2013) This suggests that legitimacy of informational exchanges is shaped by a perceiver's belief that it originates from a relevant ingroup member who has direct personal experience and is therefore qualified to comment on the particular event (Haslam et al., 1996; Levine, 1999). Importantly however, it also suggests that support is socially mediated and will interact with its content (Gallagher et al., 2014). So whilst support offered by an ingroup source often appears to be the most beneficial, the impact of availing of support from within the group where it is potentially stigmatizing is less well-understood.

The current study then will investigate student attitudes toward help-seeking from a university source, which have been shown to be highly predictive of actual help-seeking behaviors (Vogel et al., 2005). It is hypothesized that stigma of help-seeking from a source within the ingroup (i.e., the university) will be successfully predicted by the factors discussed above which have previously been shown to be influential, namely stigma of suicide, experience of help-seeking, and exposure to suicide. As gender differences have continuously been noted in help-seeking behaviors and attitudes (Andrews et al., 2001; Gonzalez et al., 2005; Vogel et al., 2011) this will also be included in our predictive model. We further hypothesize that identification with the ingroup will predict stigma of help-seeking from a university source over and above the already known predictors and demographics. Based on previous research it can be expected that high group identification will result in a lower stigma of help-seeking from formal university support services, whilst low identification with the ingroup may act as a barrier to help-seeking when the source of help is drawn from that group.

## Materials and methods

We administered a comprehensive battery of questionnaires online using a web-based interface, SurveyMonkey, which allows the collection of quantitative and qualitative data. The study received full approval from the Education and Health Sciences Research Ethics Committee at the University of Limerick (2014_06_26_EHS).

### Participants

The study sample comprised of students enrolled at the University of Limerick in the mid-west of Ireland. This population is largely comprised of Irish students, with 91% of the 14,300 students enrolled in the University of Limerick claiming Irish nationality. All registered students at the university were invited to participate in the study via an email web-link, with 693 volunteers clicking on the web-link in the first instance. The first page of the survey provided study information, assuring volunteers that participation was voluntary and anonymous, and screening questions relevant to the exclusion criteria. These were that participants had to be a current student and over the age of 18, which eliminated 33 respondents. The final sample consisted of 493 students who completed all elements of the survey, of whom 193 were male (39.1%). Participant ages ranged from 18 to 61 (*M* = 25.22, *SD* = 9.56). The majority of students that were excluded from the final sample due to non-completion did not proceed past either the information sheet (*n* = 63), or providing their demographic information at the beginning of the survey, and so did not respond to any of the measures assessing our key variables of interest. For those that completed the demographic information, One-Way ANOVA's revealed that there was no significant difference between completers and non-completers in either age [*F*_(1, 659)_ = 0.96, *p* = 0.27] or gender [*F*_(1, 659)_ = 0.74, *p* = 0.38]. Although the final sample of 493 students is representative of the total Irish third-level student population of 217,520 (2014/2015; HEA, 2015), with 384 participants required to achieve a 95% confidence level and confidence interval of 5, the participants in this study were all drawn from a single university.

### Measures

#### Exposure to suicide

Exposure to suicide was measured by two questions that were composed for this study, “Do you know somebody who has died by suicide?” and “Have you direct personal experience with suicidal thoughts, feelings or behaviors?.” Answers were in a Yes/No format with “No” given a score of zero and “Yes” given a score of one. A total score for exposure to suicide was then obtained by summing these two items, with a potential range of 0–2 and higher scores indicating more exposure.

#### Experience with help-seeking

Experience with seeking help was assessed through a single question that again was composed for this study: “Have you or somebody you know ever received professional help for any issues related to mental health?.” Responses were in a Yes/No format (No = 0, Yes = 1).

#### Stigma of suicide

Stigma of suicide was measured using the Stigma of Suicide Attempt (STOSA; Scocco et al., 2012) scale. This 12-item scale is based on Link's (1987) Perceived Discrimination-Devaluation Scale (PDD) and measures the perceived public stigma of those who attempted suicide. This particular scale was chosen as measuring public stigma rather than personal stigma has been shown to remove the potential social desirability response bias and give a more accurate reflection of internalized stigma (Griffiths et al., 2006; Peluso and Blay, 2009; Calear et al., 2011). Moreover, unlike most measures of suicide stigma, STOSA questions are oriented toward a person who attempted suicide rather than a person who died by suicide. Survivors of suicide attempts have been reported to be particularly stigmatized and often dismissed as attention-seekers, with little in the way of support offered (Sudak et al., 2008). As such this group can be seen to reflect the extent of negative attitudes and stigma amongst the general public toward suicide and suicidal behaviors. We also reasoned that by framing questions toward suicide attempts rather than the person who died by suicide underlying stigma may be revealed as the traditional reluctance of people to stray from the rhetoric of “never speaking ill of the dead” is eliminated. The 12 questions in the STOSA scale follow the semantic structure of the PDD, but investigate attempted suicide rather than depression, e.g., “Most people would treat a person who has attempted suicide just as they would treat anyone.” Responses were scored on a 4-point Likert scale ranging from 1 (strongly agree) to 4 (strongly disagree), with half the items reverse-scored. Mean scores were calculated with higher scores indicating higher levels of stigma (α = 0.85).

#### Group identification

Ingroup identification was assessed using Leach et al.'s (2008) 10-item Self-Investment scale which was used to measure identification with the individual's university. The scale design stipulates that for each item the researcher should insert the name of the group under investigation in place of “[Ingroup].” This widely used measure is comprised of three subscales, Solidarity (α = 0.84), e.g., “I feel committed to people in my university,” Satisfaction (α = 0.90), e.g., “I think that people in my university have a lot to be proud of” and Centrality (α = 0.82), e.g., “Being in my university is an important part of how I see myself,” but only the overall scale was used in the current study. Items are scored on a 7-point Likert scale (1 = strongly disagree, 7 = strongly agree), with a higher score indicating a greater level of identification with the ingroup (α = 0.90).

#### Stigma of help-seeking from the ingroup

The Self-Stigma of Seeking Help scale (SSOSH; Vogel et al., 2006) measures a person's self-evaluation for seeking professional psychological help. This 10-item scale is scored on a 5-point Likert scale ranging from 1 (strongly disagree) to 5 (strongly agree), with five items scored inversely. A higher total score indicates a higher stigma of seeking help. In the current study the SSOSH was used to predict attitudes and willingness of a person to seek professional help from the mental health service in their university if they were experiencing issues related to suicide or mental health, and the wording was adjusted to reflect this; for example, “It would make me feel inferior to ask a therapist in my university for help.” This yielded a Cronbach's alpha of 0.87, which was in line with the range of internal consistencies demonstrated in previous college samples (0.86–0.90; Vogel et al., 2006).

### Analytic procedure

#### Preliminary analysis

Pearson's correlation coefficients (point-biserial correlation coefficients for dichotomous variables) were computed in order to investigate the relationships between variables (see Table 1). These revealed significant positive relationships between the dependent variable (stigma of help-seeking) and all predictor variables other than exposure to suicide. Although a positive relationship was expected for stigma of suicide based on previous research, the direction of this relationship was more surprising for group identification and experience with help-seeking. Gender differences for study variables were assessed using a series of analyses of variance to see if there was a need to conduct analysis separately for males and females.

**Table 1:** **Pearson's correlation coefficients for study variables**.

		**1**	**2**	**3**	**4**	**5**
1	Gender^#^					
2	Exposure to suicide	−0.05				
3	Experience with help-seeking	0.14^**^	0.20^**^			
4	Stigma of Suicide	0.08	0.02	−0.001		
5	Group identification	0.10^*^	−0.01	0.01	0.24^**^	
6	Stigma of help-seeking	0.14^**^	0.04	0.108^*^	0.29^**^	0.21^**^

* p < 0.05,

** *p < 0.01 (two-tailed)*.

*N = 493*.

# *Coefficient for gender represents a point-biserial correlation given that it is a dichotomous variable)*.

#### Regression analysis

Following preliminary analysis, a hierarchical multiple regression was conducted using IBM SPSS Statistics, Version 21, in order to predict stigma of help-seeking from an ingroup source. This analytic technique allowed for the additional predictive value of our key variable of interest (group identification) to be established while controlling for previously known predictors. Variables were added in two blocks with known predictors of help-seeking entered in Block 1 (gender, sigma of suicide, exposure to suicide, and experience with help-seeking) and group identification added in Block 2.

## Results

### Descriptive statistics

**Figure 1:**
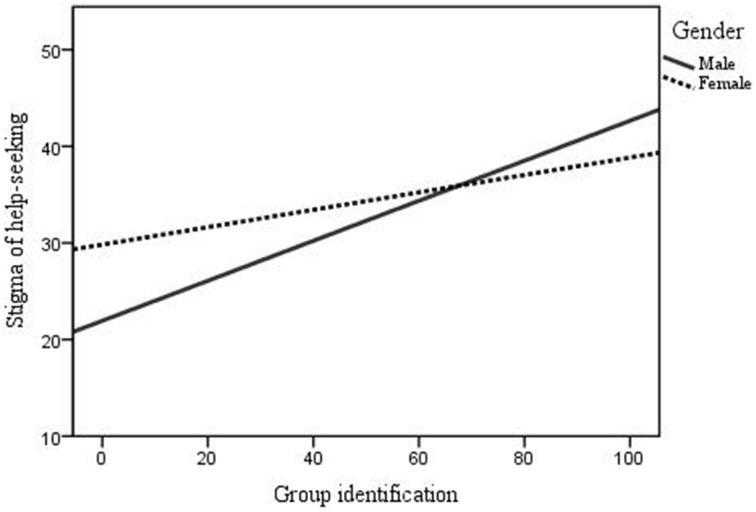
**Relationship between stigma of help-seeking and group identification for males and females**.

Exposure to suicide was high amongst survey respondents with 74.6% of participants (*n* = 368) indicating that they knew someone who died by suicide and 66.9% (*n* = 330) reporting that they had direct personal experience with suicidal thoughts, feelings of behaviors. Experience with professional help-seeking was also high; 72.6% (*n* = 358) of the sample knew someone who had received professional help for issues related to mental health. Stigma of help-seeking was prominent amongst participants, with the mean score of 33.83 (*SD* = 7.26) falling within the range that Vogel et al. (2006) class as being high (32+). Gender differences for stigma of suicide, group identification and stigma of help-seeking were explored using a series of analyses of variances, the findings of which are presented in Table 2. Although some gender differences were noted the overall trend in the relationship between our key variables of interest remained the same (i.e., for both males and females stigma of help-seeking increased with higher group identification; see Figure 1), meaning they were not treated separately for the main analysis.

**Table 2:** **Analysis of variances for gender differences in study variables**.

	**Male (***n*** = 193)**		**Female (***n*** = 300)**		**Total (***n*** = 493)**		***F***	***df***	***p***
	***M***	***SD***	***M***	***SD***	***M***	***SD***			
Exposure to suicide	1.46	0.38	1.39	0.38	1.42	0.38	1.09	492	0.30
Experience with help-seeking	0.65	0.28	0.77	0.22	0.73	0.23	8.70	492	0.003
Stigma of suicide	2.64	0.44	2.70	0.45	2.68	0.45	3.10	492	0.08
Group identification	51.30	10.57	53.46	10.28	52.61	10.43	9.78	492	0.002
Stigma of help-seeking	32.60	7.30	34.65	7.13	33.83	7.26	5.08	492	0.03

### Regression analysis

A hierarchical multiple regression analysis was carried out with stigma of help-seeking as the outcome variable. As gender, experience with help-seeking, exposure to suicide, and stigma of suicide are already known predictors of help-seeking these were entered into the model in Block 1. Group identification was then added to the model in Block 2 to establish the added predictive value of this variable once the known predictors were controlled for. Regression statistics are reported in Table 3.

**Table 3:** **Hierarchical regression coefficients for variables predicting stigma of help-seeking**.

	***B***	***SE B***	**β**	***t***	***R***	***R*^2^**
*Block 1*					0.33	0.107
Constant	15.82	2.72		5.82^***^		
Gender	1.58	0.65	0.11	2.46^*^		
Exposure to suicide	0.21	0.46	0.02	0.46		
Help-seeking experience	1.46	0.72	0.09	2.04^*^		
Stigma of suicide	4.56	0.70	0.28	6.56^***^		
*Block 2*					0.35	0.125
Constant	12.36	2.91		4.25^***^		
Gender	1.42	0.64	0.09	2.17^*^		
Exposure to suicide	0.01	0.03	0.02	0.50		
Help-seeking experience	1.46	0.71	0.09	2.06^*^		
Stigma of suicide	4.02	0.71	0.25	5.66^***^		
Group identification	0.10	0.03	0.14	3.18^**^		

* p < 0.05,

** p < 0.01,

*** *p < 0.001 (two-tailed)*.

*N = 493*.

At Block 1, the model was found to be significant, *F*_(3, 488)_ = 14.54, *p* < 0.001, with gender, experience with help-seeking, exposure to suicide and stigma of suicide accounting for 10.7% of the variance in stigma of help-seeking, and each predictor with the exception of exposure to suicide making a significant individual contribution. Adding group identification to the model in Block 2 accounted for an additional 1.8% of variance and this change in *R*^2^ was significant, *F*_(1, 487)_ = 10.12, *p* = 0.002. Together these four variables explained 12.5% of the variance in stigma of help-seeking. Each of the variables entered in Block 1 one remained significant predictors once group identification was added to the model, with the exception of exposure to suicide which remained non-significant.

Stigma of suicide was the strongest predictor; for every additional unit on the stigma of suicide scale, stigma of help-seeking increases by 4.03. This was followed by experience with help-seeking, with those who knew someone that had received professional help for mental health problems scoring higher on the stigma of help-seeking scale by 1.46. Our key variable of interest, group identification had a positive relationship with stigma of help-seeking. Every additional unit on the group identification scale resulted in a 0.10 increase on the stigma of help-seeking scale.

## Discussion

This study aimed to predict students' stigma of seeking help from their university mental health service using a combination of already known predictors in addition to a previously overlooked factor, group identification. These variables, namely gender, experience with help-seeking, exposure to suicide, stigma of suicide, and group identification, were found to significantly predict stigma of help-seeking from the university. Unexpectedly, higher levels of group identification, in this case identification with the university, were found to predict higher levels of stigma of help-seeking from the ingroup over and above the other known predictors. Thus, students who identified more strongly with their university demonstrated higher stigma in seeking help from university mental health services. These findings were not as we had predicted.

Similar to previous studies (Corrigan, 2004; Vogel et al., 2009), those perceiving higher stigma of suicide also demonstrated a higher stigma of help-seeking. For those who knew someone who had received professional mental health support in the past however, stigma of help-seeking was also higher. This contradicts previous findings showing that past experience with help-seeking is generally a facilitator of future help-seeking (e.g., Angermeyer et al., 1999; Vogel et al., 2007), but supports Rickwood et al. (2005) claims that such experience can also act as a barrier if the experience with mental health services was negative. Females were found to have a higher stigma toward help-seeking than males, which again deviates from past findings that demonstrate the opposite (Andrews et al., 2001; Gonzalez et al., 2005; Vogel et al., 2011). This may be due to the fact that the current sample was self-selected, with the possibility that only males who had an interest or awareness around mental health participated in the study.

In terms of theory, these findings add to the growing body of literature that link social identity and shared social relationships to both mental and physical health (Cohen, 2004). Social support it would appear depends on the match between group membership of the support provider and recipient, and support does not always have the same or equivalent impact (Haslam et al., 2005). We believe the results evident in this study are particularly congruent with self-categorization theory (Turner et al., 1987, 1994) which has an emphasis on the consequences of dynamics *within* groups in determining behavior. Our study provides evidence that it is those who are most identified with their university that has the most difficulty seeking help from its counseling services. Normative fit, a key concept associated with self-categorization theory (Turner et al., 1987), is an important element of the explanation for the findings in this study. This concept can be described as the extent to which the perceived behavior or attributes of group members conforms to the perceiver's knowledge-based expectations about the social meaning of group membership (Oakes et al., 1994). If a person seeking help perceives their normative fit to the group to be poor because of their problem (in this case a stigmatized mental health issue), availing of help from an ingroup source is very problematic as they are unlikely to want to expose that they are violating the perceived social norms of the group. As it was perceived stigma of suicide amongst ingroup members that was the strongest predictor of the stigma of help-seeking in this study, this concept is particularly relevant.

In terms of practice our findings suggest that help-seeking and offering support to students through university counseling is particularly challenging, not only because of the stigma of mental health issues but also because of the sense of community that being part of a university invokes. Whilst previous research has shown that identification with a particular group can be a basis for both giving and receiving social support, this study is the first to consider the role of group identification in seeking help. Shared social identities are of course associated with shared values and characteristics (Turner, 1975) and if these are values that stigmatize an issue, the ability to avail of support for that issue within the group is compromised. Importantly social identities have performative elements, they can drive the things we do as well as the how we feel and think (Walsh et al., 2015). So whilst a university counseling service has the advantage of shared university affiliation when it offers support to its students, students may be motivated to avoid availing of this service to avoid stigma within their own group. Indeed, that is what was found in the current study with students who strongly identified with their university demonstrating greater stigma toward help-seeking than those who identified less as a university student.

This leaves university counseling services with a dilemma because identification with the university also has considerable benefits. These include higher levels of wellbeing and better adjustment to university life (e.g., Bettencourt et al., 1999; Amiot et al., 2010). In order to address these conflicting processes it may be necessary for universities to reevaluate the organization of their mental health services. It is possible that counseling services off campus, offered by external agents may be more attractive to those students who are strongly affiliated to their institution; if counseling services were perceived as more independent of the institution in terms of physical proximity, embeddedness and because of their branding, students may be more likely to seek help. This is not to suggest that mental health services should be removed in their entirety from university campuses however. These are vital resources that are utilized by a vast number of students and for the most part provide an invaluable source of help and support. Rather a variety of sources may be the solution, with both internal and external options made available to students to ensure the needs of widest possible range are met.

It is important to note here that in the university where this study was conducted, counseling services are very much embedded within the university and its systems, including by proximity, by their very clear presence in all guidance offered to students, and the role they play in supporting students with academic processes (i.e., mitigation). Thus, it is important that future research compares institutions where counseling services are more or less embedded and perceived as part of the ingroup in order to further understand the practice implications of the effects we have reported here. Other limitations of the current study include the way in which participants were recruited. All of the students in our institution were emailed and asked to participate in this study. Our final sample represents a self-selected sample of this total population and as a consequence these respondents may not be representative of the wider student pool. Our sense is that students that were particularly interested in mental health issues may have been more inclined to complete the study. In addition, the population from which this sample was drawn represents a particularly homogenous group in terms of nationality and background, with 91% of all students in the university where the study was conducted identifying as Irish. Although this homogeneity may have contributed to the overall high level of identification with their university, it must also be noted that findings may differ in student bodies that are more diverse, or indeed amongst different cultures.

Future research is needed to address both this and other issues. As the effect sizes in this study were quite small, there is a need for more research to confirm our findings. This should also take into consideration additional factors that may influence help-seeking but were not included in the current study. For example, beliefs about the usefulness or effectiveness of mental health services have previously been shown to be important predictors of help-seeking behavior (Downs and Eisenberg, 2012) whilst intentions to seek help are thought to be influential here too (Schomerus and Angermeyer, 2008). Although past experience with suicide and help-seeking was measured, these questions did not go into depth or probe for the closeness of that experience, for example whether it was oneself, family member, friend, or other person. Furthermore, the current mental health status of the sample may be influential, and should be incorporated into future research. It should also be explored if students' stigma of help-seeking differs for internal and external (e.g., GP, independent counseling service) sources of help. Finally, our study was based solely on self-report measures as stigma and group identification are entirely subjective concepts and are crucial to decisions to seek help. However, this does mean that the study should be interpreted with due caution as a consequence of shared variance associated with the method, particularly as our results are correlational. Future research could usefully employ a longitudinal design to explore stigma and group identification as true determinants of help-seeking.

In conclusion, the current study found that that identification with their university may influence student attitudes toward seeking help from the university mental health service. After controlling for already known predictors of help-seeking for issues related to suicide and mental health (stigma of suicide, gender, experience with help-seeking, and exposure to suicide), it was found that students who identified more strongly with their university demonstrated a higher stigma of seeking help from this source. Possible explanations for this lie with the self-categorization theory and normative fit. This finding has potential implications for mental health service provision across higher level institutes, who may need to consider the need for a greater separation from university involvement in such services.

### Conflict of interest statement

The authors declare that the research was conducted in the absence of any commercial or financial relationships that could be construed as a potential conflict of interest.
